# A whale of a time: engaging in a war of values for youth activism in science education

**DOI:** 10.1007/s11422-022-10140-5

**Published:** 2023-01-09

**Authors:** Rachel A. Gisewhite

**Affiliations:** Center for Science and Mathematics Education, The University of Southern Mississippi, 118 College Drive, Hattiesburg, MS 39406 USA

**Keywords:** Informal science education, Erotic ethic, Phenomenology, Marine science education, Marine mammals

## Abstract

Exposure and experience with ethical dilemmas and controversial socioscientific issues provide a link to students’ lives or a pathway for sympathy/empathy and care, where youth use emotion to engage with the scenario and develop critical thinking skills to respond to ethical issues. For this theoretical paper, I focus on how informal science can be used in science classes to provide such exposure and experience, creating spaces for students to foster erotic relationships with the nature-Other and their local environment. More specifically, this paper aims to discuss how educators can use these informal science experiences, and in this case—those involving marine mammals, to find value for natural phenomena through erotic generosity and phenomenological experiences within the environment and use their knowledge and power to act responsibly.

Over the last few days, several of my friends and family members scattered across the country have reached out to see how my family is faring during the first tropical storm to impact the Gulf Coast this year. We talk about the wind and rain, and I mention that today is much warmer and more humid than in recent days. In fact, aside from the current storm conditions, we have had, in general, an unseasonably warm winter and spring in coastal Mississippi. Though still a pleasant break from the sometimes overwhelming heat of Gulf Coast summer and early fall, I have enjoyed running on the beach in shorts and a tank top for months now, even able to wade several yards into the tea-colored Gulf in search of shells and peaceful moments, or spending many hours playing outside with my children without the need of a jacket. While I enjoy the benefits of employment on a beachfront campus or living just a few blocks from the Gulf of Mexico, I use mindfulness and the nourishment of an erotic connection with the sea to stay intimately grounded in my environment—phenomenologically in my place. Plants are flowering, jellyfish are swarming, and the balmy air has yielded extreme weather conditions ripe for tornados. The National Oceanic and Atmospheric Administration (NOAA 2020) has even predicted an above average hurricane season this year. I wonder about the causes and the implications. I wonder about my children and the children in my community and if they, too, are observing the climate and their environment. I wonder about the children in other parts of Mississippi and if they get it—their role, their ability, and their power. I wonder about kids across the country and if they are learning what they need to in and outside of their science classes to help guide them on this journey of life with responsibility and care for the environment. I wonder about the value our youth, here referring to K-12 students, assign to the environment and their place within it.

You see, last year, nearly 300 bottlenose dolphins and over 100 sea turtles washed ashore along the Gulf Coast. This number of losses is three times as many as normal. For the first time since the Deepwater Horizon oil spill in 2010, NOAA (2020) declared the phenomenon an “Unusual Mortality Event,” meaning the number of dead dolphins was concerning enough to warrant an official response. The director at the Institute for Marine and Mammals Studies (IMMS), Moby Solangi, explains that we should be paying attention to mortality rates of aquatic mammals at the top of the food chain because when they’re affected, there’s a good chance the organisms in their food chain are also impacted (Snell 2019). They are, in essence, a canary in the coal mine of their environment. Before the 2010 BP oil spill, there were on average 20 dolphins (Sea Grant 2020) and ten sea turtle (STSSN 2020) deaths on the Mississippi Gulf coast per year. The drastic increase in mortality rate from last year has researchers hypothesizing that such a large number of strandings is due to exposure to low salinity waters resulting from an extremely wet winter in the southern and Midwestern United States (NOAA 2019), but also in part due to the four months the Bonnet Carre Spillway was open last year (Snell 2019). This spillway, situated in St. Charles Parish in Louisiana, can be opened to protect the lower Mississippi River valley from flooding. When open, the Mississippi River water is redirected into Lake Pontchartrain, which flows into the Gulf of Mexico. This brings fertilizers, pesticides, and other chemicals from more than half of the USA and an insurgence of freshwater that the habitat is not accustomed to. The lowered salinity has lasting negative repercussions on marine organisms, including dolphins, sea turtles, oysters, and shrimp, and creates concern for an abundance of toxic algae. This affects the fisheries, which provides jobs for thousands of people along our coast, and tourism and recreation as 25 beaches of Mississippi were closed for several weeks last year due to a harmful algal bloom (Chappel 2019). If the spillway isn’t opened and parts of Louisiana flood, residents may be displaced and in need of shelter or at risk for material damage to their homes and property. This scenario presents a war of values, yet our immediate anthropocentric concerns and inconveniences can arguably be rectified long term through care and help for our ecosystem.

The ecosystem needs help, alright. But how? Often, when I consider this question, when I wonder if I am doing enough, I think of the Bastille lyrics, “Oh, where do we begin? The rubble or our sins?” (Bastille 2013). These lyrics, sung in the British band’s 2013 song “Pompeii,” are purportedly about an imaginary conversation held between people stuck in the rubble caused by the 79 AD eruption of Mount Vesuvius in Pompeii (Smith 2019). After this tragic event, the casualties’ casts, bones, and remains were found in ash deposits and at the Herculaneum vaults, bodies frozen in action. In this dark imagery, the contorted position of the bodies found within the rubble might reveal for many a snapshot in time of a life of vice, hidden or otherwise (the “sin”). My consideration of these lyrics upon pondering the predicaments we face in our ecosystem is not directed at this or any specific historical event but toward the myriad of everyday actions or inactions we commit (our “sins”) that directly impact the environment in negative and harmful ways (the “rubble”). It is a daunting task to determine what needs to be done to live more sustainably and rectify our grievances, but it is not impossible. Perhaps the answer is that both the rubble and our sins must be addressed simultaneously. While this requires a much more extensive conversation, with the problem of informal science learning being complex and often nebulously outlined, the consideration of the lyrics begs the question: How are our youth able to make meaning and find value in various informal learning contexts? Without such moral and ethical thinking, which aids in the development of our values, students are less likely to engage actively with their environment and live a more transformative and erotic life (Luther 2013). This theoretical paper sets out to address the question of how to prepare youth through science education to be active citizens, knowledgeable and capable in the skills needed to act with mutual generosity for themselves, their communities, and the other? This paper aims to discuss how teachers can use informal science education, and more specifically here—those involving marine mammals, to guide students to find value for natural phenomena through erotic generosity and phenomenological experiences within the environment. I begin by outlining phenomenology as the philosophical underpinning, as it couples embodied experiences and consciousness. These are also essential components of eroticism, as outlined below. Additionally, I briefly discuss moral–ethical development in youth and the place for value in science education with specific examples using informal science. Through this valuation, youth can use their knowledge and power to act responsibly (Mueller and Luther 2013)—to take steps to clean up the rubble and address their own sins and possibly those of the generations before them.

## Phenomenology and erotic generosity

Outlined initially by Edmund Husserl (1970), phenomenology is the study of conscious experiences from the firsthand point of view (i.e., a peeling away of the layers of self: temporality and being), or the analysis of the relationship between the perceiver and perceived. He argues for a return to the study of phenomena in their pure state, as the things themselves, starting with how they present themselves to the immediate level of consciousness. His philosophy focuses on intentionality, how people understood a situation or experience before they began to make meaning of it, or in other words, how the phenomena come to our conscious awareness in the form of an intention. As we are conscious of objects, we are in relation to them. We perceive objects as they present themselves in the world, which comes from within, but others also perceive the objects based on their own experiences, which we experience externally. David Abram (1996) explains through this “associative ‘empathy,’” our embodied subject becomes open to other subjectivities (p. 37).

The phenomenological field is created by multiple subjectivities, including oneself. This intersubjective world, or lifeworld, is a key feature of phenomenology. The lifeworld “is the world of our immediately lived experiences, *as* we live it, prior to all our thoughts about it” (Abram 1996, p. 40, emphasis original). Although it is based on how we go about our daily lives, for an embodied subject, the lifeworld constitutes their natural being, the foundation of belief, social, or cultural systems that they ascribe to and uses to approach daily personal, worldly, and interpersonal experiences. The lifeworld is based on profound interpersonal experiences and is collectively created. However, it is also “profoundly ambiguous and indeterminate” (Abram 1996, p. 40) because how we experience a lifeworld is based on our situation relative to it.

For Husserl, phenomenology should demonstrate that practices within philosophy and the social and natural sciences are not used to interpret or give meaning to phenomena because the description of an experience or object originates with the phenomena or entity. The purpose of phenomenological theory is to clarify, not explain or give meaning to phenomena. Amedeo Giorgi (2005) argues that such clarification can provide an understanding of how we relate to the world or others, supporting constructive change because we are better able to distinguish between what is actually occurring in our lived state and what we think is occurring. Through clarification, we can live more authentically because we can bracket all of the inessential details to reveal the pure state of the phenomena or entity, our experiences, and our intimate relationship with (O)thers: both general *others* and marginalized *Others.*

Before delving more fully into this exploration of phenomenology and generosity, it is important to first clarify the meaning of this *Other.* Plumwood (2002) explains that Other historically refers to “lesser beings” that are oppositional to western rationality, culture, and philosophy. Particularly, the Other is counter to “the province of elite men who are above the base material sphere of daily life and are entitled to transcend it because of their greater share of Reason” (Plumwood 2002, p. 19). Though Others have included physically and materially “weaker” individuals or groups, modern rationalism views reason as the major factor for domination of the Other. The Other has included, for example, women, enslaved people, animals, nature, and marginalized citizens and cultures. I argue that it also consists of the ocean environment and its inhabitants.

Martin Heidegger (1962), Husserl’s student and assistant, altered Husserl’s view of phenomenology, arguing that we are part of the world through our actions, not in spite of the world. We study our actions and ourselves while considering the authentic context of our situation in relation to the world. In other words, Heidegger’s philosophy differs from the more classical phenomenology of Husserl in that it is not focused on consciousness, but on being, or being in the world through lived experiences and the interpretation and creation of personal meaning through these experiences. It is not enough for the thing to present itself because things do not always present themselves as they are. Rather, “phenomenology is seeking after a meaning which is perhaps hidden by the entity’s mode of appearing” (Moran 2000, p. 229). Part of “being in the world” is experiencing and sharing the world with others, which Heidegger (1962) describes as “care,” and others have described as “empathy” (Thayer-Bacon 2003) or an erotic connection (Luther 2013). These terms are descriptively and interchangeably used here to represent the shared phenomenological experiences with (O)thers that create a pathway for actions to benefit the (O)ther.

Similarly, Maurice Merleau-Ponty’s (1962) phenomenological view is based on perception, but in his case, more *embodied* perception over exclusive rationality. Merleau-Ponty explains his “phenomenology of origins” (as cited in Abram, 1996):

All my knowledge of the world, even my scientific knowledge, is gained from my own particular point of view, or from some experience of the world without which the symbols of science would be meaningless. The whole universe of science is built upon the world as directly experienced, and if we want to subject science itself to rigorous scrutiny and arrive at a precise assessment of its meaning and scope, we must begin by reawakening the basic experience of the world, of which science is the second-order expression…To return to things themselves is to return to that world which precedes knowledge, of which knowledge always *speaks*, and in relation to which every scientific schematization is an abstract and derivative sign-language, as is geography in relation to the countryside in which we have learnt beforehand what a forest, a prairie or a river is. (p. 36, emphasis original)

Human consciousness is a result of worldly things, ideas, experiences, and for Merleau-Ponty, perception is experienced bodily through worldly experiences at a prereflective level. For the perception to be gained, one must reflect on the experience. Reflection, however, is based on the use of language, which Merleau-Ponty argues tears us away from the unique qualities of the experience: To name something or put something into words establishes a meaning based on a representation or categorization (Ozman and Craver 2011). In considering this point, we realize that perception is not a pure abstraction, and it does not always lead to truth. Perception, however, is the foundation of truth-seeking when experienced and coupled to reflection. It is influenced by our temporal and spatial place in the world and by our intentionality, the interaction between subjects and things of the world. Merleau-Ponty began using the term *flesh* to describe this interaction. The flesh begins to get at the idea of *sensuality*, or relating to the senses, which is an important component for phenomenology in science education, as I will discuss later.

A key element of Merleau-Ponty’s philosophical outlook is maintaining the relation of being in the world, or as he sometimes refers to as flesh of the world, in which self cannot be separated from the world. We only have one world, which presents itself to us, and which we experience primitively as a child before we are influenced by the “distortions” of science. Further, this primitive relationship with the world is one lived within it, not as an outsider or observer of things as science promotes. In essence, the idea of objectifying, categorizing, and labeling in science has significantly gone astray from a more enlarged nature of science. This reduction to the primitive world of a child is Merleau-Ponty’s call for us to “participation in the here and now, rejuvenating our sense of wonder at the fathomless things, events and powers that surround us on every hand” (Abram 1996, p. 47). Living bodily within this world is where we find our true faith. Merleau-Ponty explains, “it is this unjustifiable certitude of a sensible world common to us that is the seat of truth within us” (1968, p. 11). Interestingly, Merleau-Ponty also discusses the truth within us when referencing our relation to the Other:

The experience of the other is always that of a replica of myself, of a response to myself. The solution must be sought in the direction of that strange filiation which makes the other forever my second, even when I refer to him to myself and sacrifice myself to him. It is in the very depths of myself that this strange articulation with the other is fashioned. The mystery of the other is nothing but the mystery of myself. A second spectator on the world can be born from me. (1973, p. 135).

Perhaps, then, under this logic, we can develop empathy and care for the Other, and phenomenology will likely influence an entirely different way of being in science education, including the non-human Other, through the adoption of the Other’s perspective when we return to the primitive world, experienced of the flesh.

Merleau-Ponty’s friend and contemporary, Simone de Beauvoir (1948, 2011), extends these ideas to human relationships in her theory of ambiguity, which provides a mirror for us to see the Otherness of ourselves. We are ambiguous by nature. We are also gendered and a product of history, culture, and society. Though we are very much aware of the mortality of our human situation and strive in many ways to escape ambiguity to establish a mind/body dualism (and avoid vague or negative connotations), Beauvoir (1948) argues that we can achieve transcendence if we assume our ambiguity. Through ambiguity, we have the wherewithal and strength to live ethically and morally. We gain wisdom from understanding and striving for the project to accept freedom and responsibility of self and desire freedom of Other(s). We acknowledge, when applicable, the need to release the shackles of oppression. In other words, part of Beauvoir’s theory of ambiguity is to use generosity to try to understand the situation of the Other.

Closely aligned with Beauvoir’s work, the premise of my phenomenological erotic ethic (Luther 2013) is people assuming their natural ambiguity to consciously grant freedom to self and Others. The erotic life requires us to live embodied, where this phenomenological experience in the flesh blurs the boundaries between self and Other. This allows us to realize that the Other’s subjectivity is not something to be fearful of or oppress. In other words, the erotic provides openness to the Other through the flesh. The openness of the flesh grants the opportunity and responsibility for the moral valuation of the Other, providing a need and a place for generosity. In this acknowledgment of the possibilities of the Other, we can act for the freedom of the Other. This action is erotic generosity.

Generosity, or giving of oneself for the sake of the Other, is necessary to keep us from objectifying the Other and evolves as we allow ourselves to be open to the world, to participate within the world while acknowledging freedom of self and Other. Erotic generosity is only possible through embodied, phenomenological experiences through the flesh, in which we can identify aspects of ourselves in the Other without romanticizing the Otherness of the Other (Luther 2015). Generosity is essentially to seek the freedom and happiness of the Other, rather than to dominate or enslave the Other. Erotic generosity evolves as we are open to the world, and we participate within the world without controlling or objectifying the Other.

As ambiguous, embodied, and erotic people, we can use generosity to grant freedom to Other(s), engage in meaningful relationships, and find joy and love in self and Other(s). Before addressing how the erotic ethic and phenomenological experiencing can promote moral and ethical thinking in informal science settings that can lead to valuation and the potential for action, it’s important to briefly consider children’s moral development.

## Finding the high ground

Nel Noddings explains that “to behave ethically is to behave under the guidance of an acceptable and justifiable account of what it means to be moral” (2013, p. 27). Generally speaking, ethics and morality deal with the notions of “should” and “should not,” considering both individual action and feeling (morals) and relational associations and behaviors established by societal norms and influences (ethics). These terms are often used interchangeably by theorists, as they are in this paper. Values, on the other hand, are what we consider to be worthy in a way that also motivates individual action or frames individuals’ attitudes. Ethics consider the values people live by and help us determine which should be the case and which should not.

Youth begin to develop ideas of “should” and “should not” at a young age, even as they are toddling, through experimentation with moral dilemmas. Young children initially develop their voice of conscience from a fear, initially of punishment from parents, then teachers, then self (Kohlberg 1976). Jean Piaget was considered among the first to investigate moral thinking in children and explains that children’s moral development is determined by cognitive development and influenced by social interaction with their peers. Preschoolers see moral dilemmas in black and white: An act is either wrong or right. As children grow older, they are better able to move from objective responsibility, how much harm they've done, to subjective responsibility, their intentions to harm (Piaget 1932). Lawrence Kohlberg (1975) extended this notion of a progression of moral development constrained by cognitive development. According to Kohlberg, youth must pass through stages of moral development, where they cannot understand a higher level of morality until they can fully understand their present level. In this way, some youth may plateau at a certain level for long periods. Children can move upward in their progression of moral development through regular exposure to reasoning at a higher level, engagement in rule-making and questioning, and the use of real-world examples. Further, children are faced with moral dilemmas more frequently in their teen and young adult years. Through these experiences, youth are challenged to choose between two or more actions based on evidence and consequences, and with increased experience comes a greater opportunity for growth.

This is a simplified version of moral development. Of course, how youth develop moral reasoning is dependent on many factors, not solely on age. Culture, religion, relationships and interactions with others, parental influence, motivations, and values education are among the various factors that can influence when and how children develop value clarification and moral reasoning. Further, scholars of morality education differentiate between an ethic of care, concerning relationships and responsibility, and an ethic of justice, which is a more formal determination of rights and rules (Mortari and Ubbiali 2017). A caring ethic suggests that a caring person is one that “regularly establishes and maintains caring relations; it isn’t enough to have caring as a motive” (Noddings 2015, p. 411). The focus here is on the *action*. Viewing these understandings of moral development through the lens of the erotic ethic reveals how youth can demonstrate a desire for protection and conservation through erotic generosities, *acting* for the sake of the Other because of the care found in the valuation of the relationship and the Other.

More specifically, while we may work hard to be more ethical and compassionate people, because we are subject to the uncontrollable conditions of human existence, the erotic ethic cannot definitively offer what is right or wrong, or who is to blame in certain situations. However, part of being ambiguous is not knowing how our past or current actions will affect our future possibilities. Though we have no specific gauge for moral freedom and integrity, if our freedom is reliant on the freedom of the Other, we will always act in a way that offers the possibility of moral freedom. In other words, we always have an impression of whether our action will lead to moral freedom, particularly in considering whether we are treating the Other as a subject or an object. In *The Ethics of Ambiguity* (1948), Beauvoir notes that though it is our responsibility to always act morally for the sake of the Other’s freedom, it is in our intention that we gain freedom as well. Despite our best intentions, we cannot act for the Other, only with the Other. Therefore, it is relationality that makes this erotic ethic different. Ultimately, we can know that we act more compassionately and morally if we treat the Other as a subject worthy of freedom and care, valuable in its own right, and a moral agent.

### Youth activism

It is important to note that the intention of this work is to avoid “indoctrinating” philosophies. Rather, the focus is on a *quality* science education, where students are ideally taught and encouraged to be critical thinkers, not presented biased information and trained to achieve a set end goal. Hudson (2001) writes, for example, “at times there have been efforts to ‘dumb down’ the existing scientific underpinnings of environmental knowledge as a means of advancing an agenda that depends on an unsustainable, resource-extractive approach to economic development” (p. 284). This bias of information is particularly detrimental as students are encouraged to act for an issue that has been presented as skewed, often through a crisis approach (Mueller, 2009). Instead, educators ought to focus on teaching students the skills and knowledge needed to carefully analyze issues before taking actions and then reflect in some larger way on those actions (Hodson, 2011). Students should not be encouraged or guided to act on issues for the sole benefit of the educator’s agenda and without careful consideration and reflection. Youth activism learned in the ways through indoctrination and biased presentation could actually lead to frustration and disempowerment (Connell, Fien, Lee, Sykes, and Yencken, 1999) rather than giving students a sense of competence and enthusiasm for action, which is the goal. A quality science education can promote this goal through ecological sustainability and conservation efforts, educating students on the affected Others (human and non-human), and empowering youth to become involved citizen stakeholders and advocates for evaluating socioscientific issues of ecojustice and work to revitalize the commons. The investigation and integration of values in science education for enhanced moral and ethical thinking and action addressed here does not condone indoctrinating pedagogy, but rather encourages one that gives youth the knowledge and skills they need to make decisions of action based on moral and ethical considerations and an understanding of one’s own valuations.

### Values in science education

Sea goers and coastal inhabitants have been studying the ocean informally since the beginning of human existence, stemming from relationships built on sustenance and wonder. In science education, informal learning environments are often considered to be places like museums, zoos, and aquariums, but informal science education (ISE) occurs in the cross section of natural or scientific phenomena and the lifeworld of the student. It is defined as science-related activities that occur outside of a formal school setting and are not associated with school curriculum or for school use (Crane et al. 1994) and includes, but is not limited to, learning science through museum and aquarium experiences, media (e.g., newspapers, books, television, Internet), social interaction with friends, family, and community (Dierking et al. 2003). The potential benefit of informal learning spaces is compelling for increasing scientific reasoning, which is an important goal of informal learning environments indicated by the National Research Council (2009).

However, discussions of morality and ethics are also a natural fit in science education settings. As students learn about the Nature of Science (NOS), they begin to understand that science has a unique set of values that include, among other things, research integrity and the ethical reporting of scientific findings (NGSS, 2013). The meaning of the term *value* can be influenced by various factors, such as religion, economy, or politics. For the purposes of this paper, we consider it at a basic level that it acknowledges something as worthy, inspires action, and develops personal attitudes. Scientists make a value judgment in the various components of their inquiry cycle, from defining concepts to quantitative measures (Schroeder 2020) to scientific communication. These judgments stem beyond looking at evidence and data for decision-making. Some philosophers of science argue that it is the duty of scientists as moral agents to consider the consequences of all empirical claims made from their research and that within the scientific community and make value judgments accordingly. However, while some argue that scientists defer to the public on the meaning of values (e.g., Douglas 2009), others suggest this task fall to the hands of a democratically legitimized institution (e.g., Betz 2013). In a general sense, unless funded by a political entity or government agency, we can assume scientists make value judgments as would a private citizen (Schroeder 2020). To ensure that their value judgments are legitimate and responsible, Kevin Elliot (2017) suggests that scientists must be transparent with their work, representative of major social and ethical concerns, and engaged in critical analysis by multiple stakeholders. In addition to this regular influence of values on science, many scientists are picking up the torch from such scientists as Rachel Carson and Theo Colborn, who actively called for the public to act against environmental pollutants that impact human and animal health by appealing to their value systems.

As much as value systems can, at first glance, appear subjective, countries’ values often reflect those of the individuals living there (Fischer and Poortinga 2012). Additionally, through the scientific process, bias can be reduced or eliminated through peer review and transparency. In situations when values conflict, like, for example, in consideration of indigenous relationships with animals via hunting versus the valuation of the animal life, we may be forced to make a choice. This frequently occurs through an often unconscious ranking process. Interestingly, human valuation is inherently considered positive to the individual, so this ranking process does not exclude options but simply shows which is valued more (Hitlin and Piliavin 2004). In essence, these are the lessons we can teach our youth through science education: Science is constantly influenced by values in an effort to conduct and promote responsible scientific investigations, but subjective bias is mediated through scientific communication with various stakeholders and peer review; and that through enhanced moral/ethical thinking and scientific literacy, value judgments and actions can be made through value ranking, where youth, their teachers, and community members can decide which values are more important or necessary for action depending on a holistic view of the situation.

Science educators have been using socioscientific issues (SSIs) as another pathway for including value systems and the development of moral/ethical reasoning. Using controversial socioscientific issues provide a link to students’ lives or a pathway for sympathy/empathy and care, where students use emotion to engage with the scenario and develop critical thinking skills to respond to ethical issues (Sadler and Zeidler 2004). Further, understanding and helping youth to form values effectively is important, because “they regulate the ways in which a learner’s/teacher’s cognitive skills and emotional dispositions are aligned to learning/teaching in any given educational context” (Seah and Andersson 2015, p. 169). This is evidenced in the type of scientific literacy developed through the use of SSIs, termed *functional scientific literacy* by Mueller and Zielder (2010), which “includes moral–ethical inquiry as a part of the larger process of becoming informed and participating more fully in community decisions, and school science is a microcosm of the larger worldly domain” (p. 107).

What happens then if youth are taught and encouraged to be critical thinkers within informal science settings through a phenomenology of place? In these places where informal science can be found, educators and community members can lead youth through embodied experiences that encourage them to learn the skills and knowledge needed to carefully analyze issues before taking actions and then reflect in some larger way on those actions (Hodson 2011). It stands to reason, then, that in these phenomenological experiences, youth also access and develop their value systems. This coupled with increased content knowledge and skills, youth are in a better, more informed position to act for the preservation and conservation of place.

## A place for a phenomenology of place in informal science education

Phenomenology of place is where students can connect erotically with the phenomena of the natural world in their places (Luther 2013). Similar to place-based education (PBE), a phenomenology of place also grounds content knowledge and science learning within the experiential, embodied learning and encounter with phenomena (Smith 2002). Also like PBE, utilizing a phenomenology of place is a way to move away from traditional science, as focused on in STEM curricula in schools, and teach science content in informal settings with a focus on citizen thinking. However, phenomenology of place extends beyond this with an intentional focus on moral/ethical thinking and generous action. In other words, it teaches science content while promoting moral and ethical thinking such that students can afford nature equivalent moral considerations in their community. While honing moral and ethical thinking, students also build erotic relationships with the Other through phenomenological experiences (Luther 2013). These relationships provide the foundation for caring, sustainable, and generous actions that promote freedom and conservation. Further, through a phenomenology of place, students are more capable of tackling ecojustice issues, because discourse and authentic, embodied activities challenge them and make them aware of what they think regarding certain issues and their own epistemology. With the addition of scientific discourse to these phenomenological experiences, the students and their teachers can assume a scientifically literate eroticism (way of being). Engaging in the fluid proficiency of this epistemology will allow them to recognize themselves as a knowledgeable member of the community regarding aquatic ecosystems. This literacy/episteme is what grants educators and students the type of skills necessary to act for ecojustice within their community.

With such an opening, and as youth gain an understanding of the marine environment in their informal science settings, they are more likely to care for it and take action to protect the ocean and the marine environment. This is evidenced by the numerous examples of children across the country caring for local aquatic environments through participation in beach and river cleanup days, organizing recycling programs in their schools, and children walking the beach in the early morning to rescue sea turtle hatchlings that have lost their way. If the students are building relationships with natural environments that evoke a sense of responsibility and passion to protect, care, and act for that environment, then in that action, they will, in turn, continue to learn about that environment and strive to make more ethical choices in their lives for the sake of the Others. These actions are self-perpetuating, and thereby a more sustainable way to engage in scientific discovery and conservation than that of a curriculum that focuses on creating the next generation of scientists through mental training and scientific orientation.

We can develop a sense of empathy for others if we can bodily situate our lifeworld in relation to other individuals, communities, and even non-human others that help create our common field. Some eco- and evolutionary psychologists suggest that humans are born with an innate emotional attachment to the natural world as a result of genetic encoding that can be traced to our times as hunter and gatherer (e.g., Han 2010), and this innate emotional attachment can naturally lead to empathy for Other. It is important to note that this is not specifically linked to marine and other aquatic environments, though there certainly is evidence to suggest a powerful and erotic human–ocean relationship (e.g., Kuo, Barnes, and Jordan 2019). There are many reasons why it is important to focus on marine environments in ISE, including, for example, that people are relatively unknowledgeable about the ocean environment (Ocean Project 2009), and yet it covers more than 70% of the planet, influences our weather and climate, and produces over 50% of the oxygen we breathe. For the purposes of science education, the integration and implementation of marine science education provide an opportunity for more authentic scientific inquiry experiences, and a more cohesive understanding of natural systems, along with a way to create relevant socioscientific and justice connections. Youth struggle to understand the ocean on a holistic level or the aquatic ecosystems that connect them to the ocean (Ballantyne 2004), which demonstrates a need to include more ISE opportunities that investigate physical, chemical, geological, and ecological aspects of marine science.

For this paper, I focus on how exposure to marine mammals in ISE specifically provides a space for youth to grapple with their values with the potential for action. The choice to focus on marine mammals is twofold. First, interactions with these charismatic organisms have been shown to positively influence well-being and environmental connectedness (Yerbury, Boyd, and Weiler 2021). Researchers in informal science settings have also documented increased positive emotional responses, like empathy and wonder, when participants are exposed to documentaries and presentations about big charismatic animals (e.g., Barbas, Paraskevopoulos, and Stamou 2009). The second reason arose through interaction with youth across K-16 over many years of teaching marine science content of various disciplines. Over the years, I observed the spark of interest my students exhibited over talk of whales, orcas, and dolphins that was less likely to occur in the discussions on the Coriolis Effect or the carbonate compensation depth, for example. As such, marine mammals serve as an entry point to ethical considerations of the ocean environment, where youth first engage with what interests them and then learn through the science content the importance of the other aspects of the marine system that are relevant to keeping these organisms alive and their population thriving. In other words, using anthropocentric value theory as a means to capture student interest, then through opportunities afforded through ISE to learn the science content, experience authentic science and natural phenomena, and engage youth in moral–ethical conversations, youth can develop an erotic relationship with this nature-Other and experience a shift to non-anthropocentric axiology.

### Positionality of the author

A final, more personal reason for choosing marine mammals as a means to engage youth in value ethics is a direct result of living on the coast of the Gulf of Mexico. I have not always lived near an ocean, though my relationship with it began long before taking a professorship of science education on a Gulf Coast college campus. I grew up in the Rust Belt of northeast Ohio, an eight-hour drive from the ocean. I was fortunate enough to make that eight-hour trip to the beaches of New Jersey every summer with my family, where my love of the ocean began. Because of this love, when it came time to apply to colleges, I chose to major in Marine Science. I had a great undergraduate experience. I took field trips to the Everglades, the coasts of the Carolinas, and the Gulf of Mexico. I traveled on marine science research cruises with scientists, collected samples, processed data, and presented my research at a regional and national conference. I made the most of my bachelor’s degree, but it wasn’t always easy. Many of my peers in the marine science program decided to major in the marine sciences because it was an important and common part of their lives, many having grown in up coastal cities such as Charleston, Virginia Beach, and Miami. They had a clear advantage over me—I thought—already knowing so much more about the ocean than I could have dreamed, just from spending part of my summers there. In my own life and through my research, however, I began to see how intimately connected the ocean is in all of our lives as I started to understand the relevance in my own life. Yet, through these experiences, I became inadvertently aware of the neglect of marine science education in science education, particularly in landlocked states and non-coastal areas, like where I grew up. This led me to a graduate path on marine science education. Through my experiences with the ocean and learning marine sciences, as well as those of a female, first-generation college student with two STEM degrees, it has become my quest to strive for justice and equity in STEM, my community, and the environment, and inspire the next generation of teachers and active community members.

In this place I call home along the Mississippi Gulf Coast, it seems as though not a month goes by without headlines of stranded dolphins or sea turtles, closed beaches due to harmful algal blooms, consequences of tropical storms and hurricanes, a dangerous influx of freshwater or harmful pollutants from upriver. My own students have on more than one occasion needed to skip class because the roads between their home and campus were flooded. The debris on the beach following Mardi Gras parades and Fourth of July celebrations is overwhelming. And yet, we have at times been surrounded by dolphins on boat trips out to one of the barrier islands. They are curious and playful, and I want to do more than hope that our youth can experience that level of awe over dolphins at some point in their lives. I am arguing in a more general sense that it is necessary for youth to foster erotic relationships with nature phenomenologically in their place and use these experiences to ground value judgments and actions. More specifically here, the anthropocentric perplexities surrounding marine mammals serve as an opportunity for moral–ethical thinking and consideration of values. Some of the examples below were initially inspired from local issues, either to my current residence or my childhood home near a now-closed SeaWorld and evolved through further research on these topics. Through a similar process of scanning local news for environmental concerns, other science educators can encourage phenomenological experiencing for generosities in their own place.

### *Damned* if you do, *dammed* if you don’t

My local marine environment has suffered in the last few decades due to decisions made from competing value systems. Severe flooding in 2011 prompted Louisiana to open levees at several points along the Mississippi River, introducing more freshwater into the system than naturally occurs. The result was a decline in oyster production and the blue crab industry. In more recent years, turtles and dolphins have been found in the Mississippi Sound with skin and eye lesions, which is consistent with freshwater damage. When authorities opened the Bonnet Carre Spillway, trillions of gallons of fresh water flowed into the Mississippi Sound over a long period, changing the salinity from an average of 18 parts per thousand (ppt) to as low as 2 ppt along the coast. This change in salinity is drastic and fatal for many marine organisms, as evidenced by the decline in the speckled trout and redfish populations. Coastal populations of dolphins like the ones found in the Mississippi Sound, however, are territorial and not quick to leave their coastal habitat even when the salinity dips to dangerous levels. The influx of freshwater also brought in large amounts of silt, mud, clay, fertilizer, and potentially invasive species from sources upstream. To put the impact into perspective, only 91 dead dolphins were counted the year of the BP Oil Spill, one of the worst environmental disasters in history. In other words, the Bonnet Carre Spillway opening has been more damaging to dolphins than the BP Oil Spill (Broom 2019). In addition, the impact on marine organisms has invariably impacted fisherpeople whose livelihood depends on their catch and is an important part of the coastal economy.

The spillway was built in response to the great river flood of 1927 and is designed to alleviate pressure on New Orleans’ levee system. This is important because approximately one million people live in the area of southeast Louisiana the spillway is designed to protect from flooding. If the Bonnet Carre had not been opened in early 2020, many people would have lost their homes and had to go to shelters or stay with friends and family during the flooding. This was far from an ideal solution at the beginning of the COVID-19 pandemic, particularly in an area hit hard by the virus early on. In this example, those with the power to make formal decisions on the fate of the spillway had to consider, among possible others, the economy, the safety and welfare of the human population, the pandemic, and the marine environment. In this war of values, anthropogenic interests won favor, and the Bonnet Carre was opened. But what if we used these examples, and others within informal science contexts, to expose youth to the *Other*ed implications of our actions? In this scenario, perhaps a different decision would have been made or conversations favoring a shift to a more sustainable and marine environmentally friendly solution to our flooding issue if our society was better educated on the innate moral worth of cetaceans. Without these types of conversations, will youth be able to determine what erotic generosities are possible for the dolphins in this area, for example? It is unlikely that teachers are even addressing that in this case, the erotic generosities were bestowed upon those human Others that live in the lowest parts of the city of New Orleans. These people are *other*ed, in part, because of the difficulty a storm imposes on their lives due to the location of their home, the infrastructure that determines the safety of their homes and families, and the decisions that need to be made regarding risk of exposure to COVID-19 if sent to a storm shelter.

### On the case of seaworld

While part of this conversation is how informal learning environments and experiences can best communicate the role of science in society (e.g., Pellegrino 2012), what is missing in these investigations is the critique of those informal learning environments, like SeaWorld, for example, whose agenda is set up for making money from entertainment, where education seems a secondary or lower priority. Consider whether or not these places should be used ideally to promote moral and ethical valuation of the science content and exhibit animals. The documentary *Blackfish* was released in 2013, controversially drawing attention to marine mammals kept in captivity, both for their emotive and cognitive abilities, but because it highlighted the ethics of show animals or those kept in captivity for economic purposes. To be clear, this paper does not argue against the benefit of institutions that care for wild marine mammals that have been beached, injured, or for some reason cannot medically return to the wild. Like me, many of the SeaWorld trainers in the movie grew up as kids in the middle of the country, far from oceans. After attending a show at SeaWorld, they were hooked on the idea of becoming a trainer, stating in the movie that “just seeing a killer whale is breathtaking,” “being in the presence of killer whales is inspiring and amazing,” and “when you look into their eyes, you know somebody is home, somebody is looking back.” One trainer claimed that their experience as a trainer provided them with a relationship (with the whale) like they had never had (Cowperthwaite 2013).

In the wild, orcas live in complex matriarchal societies, in which females live some 50 years, 20 years longer than males, and the offspring never leave the mother’s side. Like other cetaceans, they are highly social and use their own unique languages within their family or social circle, where no two families exhibit the same pattern of clicks and whistles as another. These social and family circles are tight-knit where cultural traditions are passed down (Whitehead 2008) and youth learn how to navigate the waters through learned behavior. They express their emotions within their social circle, even through bereavement as orcas have been observed cradling their injured offspring between them or carrying their deceased infant for more than 2 weeks (Marino et al. 2020). In addition to having a brain size and structure that indicates high intelligence, observations of orcas reveal that they are highly emotional and have awareness of self (Marino et al. 2020). In captivity, however, they are changed. Many orcas live less than 10 years in captivity (Jett and Ventre 2011). Their dorsal fin is the tallest of all cetaceans, not supported by bone, but rather a collagen. In the wild, a collapsed dorsal fin is extremely rare, only occurring in those orcas that have experienced trauma, are sick or old, dehydrated, or exposed to other stressors. In captivity, nearly all male orcas and some females have collapsed dorsal fins (Alves et al. 2018), leading to speculation about the cause, including their unnatural diet, lack of exercise, or the other various stressors they face in their tanks. In fact, they experience stresses associated with socialization, sensory deprivation, confinement, boredom, and lack of control (Marino et al. 2020). Each of these stressors exhibits a range of behaviors not typically seen in the wild, including rejecting offspring or refusing to nurse, hyperaggression, refusing to eat, banging their heads against the tank, or chewing on the gates, to name a few. Taken from their families at a young age to place in zoos, aquaria, and places like SeaWorld, the broken mother–offspring bond results in a loss of learned wild behaviors needed not just for survival, but for thriving. Placing orcas in tanks with those from outside of their natural social circle is confusing, particularly as they cannot communicate beyond displays of behavior and may exhibit differing cultural or biological traits. They swim hundreds of miles a day in the ocean, but because of their limited mobility in captivity, they can often be seen floating in their tanks almost lifeless for hours at a time. The differences between wild and captive orcas abound. Using documentaries such as *Blackfish* and critical discussions on keeping marine mammals in captivity compared to their lives in the wild can not only teach students science content but will surely benefit students and facilitate the process of establishing value for marine mammals in their own right. This provides an opportunity to act with erotic generosity for orcas in captivity in meaningful ways, including, for example, engaging in conversations or even investigations with scientists regarding the orca health and well-being, writing letters to policy-makers for improved conditions, or an audit of personal or family activities that support similar negative consequences in animals.

Beyond the many issues to consider for the orcas, let us go back to the issues of these informal science centers. SeaWorld miseducates and misinforms the public by taking advantage of the high value society places on marine mammals and normalizing to new generations of youth that it is ok to keep whales in captivity—exploiting orcas for multi-million dollar corporate gain. In these types of environments, if the emotional arousal and valence are experienced during informal science settings, it is a significant predictor of successful short-term (Staus and Falk 2017) and longer-term learning outcomes (Staus 2012). Further, environmental values are also a significant predictor of short-term learning outcomes. Participants with a more proenvironmental value system experience stronger negative valence, or displeasure, than those participants with a more anthropocentric value system (Staus and Falk 2017). It stands to reason that emotional arousal and valence can still be experienced if these examples are used within science classrooms through critical conversation and scientific discourse, moral reasoning, and authentic phenomenological experiences, thereby increasing short- and long-term learning outcomes of science content.

### To hunt a whale

As a means to analyze this notion that value development can occur through phenomenological experiencing of marine mammals further, consider the ideological conversations around whaling. There are two major camps here: those that are anti-whaling and those in favor. Anti-whaling nations view whales as organisms that have innate moral worth, while prowhalers see whales as a natural resource similar to fishing. One of the biggest players in this hunt is Japan, a country that historically whaled for cultural reasons and then for sustainability after World War II. It is important to note that they are not the only prowhaling nation, as Norway and Iceland have also supported continued whaling, but Japan has over the last few decades been in hot water over their whaling practices. In 1986 all International Court of Justice (ICJ) members agreed to a hunting moratorium in order for whale populations to recover from the brink of extinction, an issue more worrisome for whales due to their slow reproduction rate. The hunting moratorium provisioned a loophole, however, for countries to continue the hunt for scientific purposes only. Since 1987, Japan has caught between 200 and 1200 whales annually (IWC 2021). In 2014 the UN’s ICJ ruled for Japan to stop their scientific whaling program in the Antarctic due to questionable scientific practices. During the ruling, Japan’s counsel argued that the ICJ could have alternative understandings of science/nonscience, and that anti-whaling nations opposed their practices for political or cultural reasons, cloaked “in the lab-coat of science” (Naranjo 2014). The issue for the ICJ was the reality of Japan’s scientific practice, which was based on the desire for certain catch sizes rather than the opposite, evident because much of the whale meat harvested through scientific practices ended up on the meat market. In short, their methods for catching, killing, and treating whales for scientific purposes did not match the objectives of their scientific plan. The anthropocentric viewpoint reigns in this conversation of economy, and marine policy choices are influenced by values. For example, in this scenario, humans are obviously positioned as supreme to these megafauna, whose moral worth does not appear to be considered nearly as much as their economic worth. Additionally, a major question to ask is what constitutes *science* for the ICJ, and does Japan meet their description of scientific practices? Notwithstanding the cultural history of whaling in Japan, non-lethal methods of studying whales also exist, which make the hunting of whales for scientific research not only inhumane but obsolete (Waugh and Monamy 2016). This example is prime to teach youth about cultural relevancy in science education; a critical reflection on the practices of whaling could support a higher degree of reflective practices in learners by questioning the cultural practices that may not make sense in the current environmental contexts.

To delve deeper, consider how in December of 2018, Japan announced their formal withdrawal from the ICJ, which would allow them to resume whaling in the exclusive economic zone (EEZ). They also withdrew from the International Whaling Commission (IWC) in June of 2019, arguing that they could hunt whales sustainably. Sustainably, however, does not mean ethically or with generosity. Many traditional whalers use the surround-and-drive method, in which several boats surround the target whale and drive it to shore. Harpooning whales, another traditional method for whale hunting, results in a long and painful death. One study revealed that during Japanese whale hunts, whales took on average 10 minutes to die, with some taking as long as 25 minutes (Gales, Leaper, and Papastavrou 2008). Even if they use the more modern methods of explosive grenade harpoons, which produces an internal explosion upon penetration that is designed to kill the whale within seconds. This assumes ideal sea conditions, little movement of the boat, and perfect marksmanship. When the harpoon does not kill the whale instantly, whalers have to turn to other methods to finish the job that can take much longer. One argument from prowhalers is the question of how this is different from factory farming, aquaculture, or deer hunting, for example. This could lead to an entirely new conversation on animal ethics, which we will not explore here. For the purposes of this paper, the answer lies in the understanding that all cetaceans display characteristics of emotion, high cognitive function, and the importance of their social structure. Another argument lies in Cartesian thought, which suggests that cetaceans may not feel pain to a significant degree because they cannot communicate that pain to us (D’Amato and Chopra 1991). Whether or not they have evolved to communicate their pain to us is ridiculous at best; we have yet to master whale cries in order to do the same, making this argument null. We further *other* these cetaceans in the belief that it is not enough proof for *us* that whales have sophisticated communication among their own species. Their physical presence provides sufficient evidence of their pain. In most whaling methods, the whale is driven from its pod or killed in front of its social circle, sometimes exposing them to the cries of agony, a lower monotone than its normal pitch (D’Amato and Chopra 1991), as the whale’s life ends. As they are hunted, sometimes for hours before they are killed, they experience fear and stress. They suffer—something researchers are quite confident in.

Before moving forward, it is important to acknowledge that whaling is a pivotal example for value and erotic ethics in the science classroom, particularly because it does not fit into a “one-size-fits-all” solution with respect to the relationships marginalized groups of people have with the land. For example, the Alaska Natives worldview includes a reciprocal relationship between humans and non-human persons, or animals, which are believed to have souls, social and familial structures, and their own knowledge. They view whaling as a social relationship, an opportunity for meat sharing and subsistence, and have respect for the animal that provides this for them (Ikuta 2011). This is a single example of many that demonstrates a need to carefully consider and challenge the notion that Western science or ways of knowing is “right.” Many native people and other marginalized groups whose understanding of and relationship to the natural world is discounted in favor of Western views already practice something akin to an erotic ethic with the land and its inhabitants. For example, they care for the animals and plants that they will later sustainably harvest as a community for sustenance, which demonstrates a mutual erotic generosity. The consideration of their marginalized knowledge is a key component of knowing how to protect the natural world. While this is a much more extensive conversation than fits the scope of this paper, we can briefly consider how this fits within the context of eroticism and values in science education. An erotic ethic for the ocean includes marginalized marine science knowledge, which connects youth to their communities and the ocean, and in this case, marine mammals, in authentic ways. If we allow our students to favor only Western science, then we silence the people in those marginalized groups and discount their ways of being in and knowing the world. Perhaps then their moral–ethical reasoning and value development for cases such as these should necessarily include a consideration of the marginalized groups. Through embodied, phenomenological experiences, interaction with non-Western or native groups, and an openness to an erotic relationship with the Other, youth can grant generosities by avoiding silencing, or even give voice to, traditionally marginalized groups and embrace their own marginalized knowledge.

### Rights for cetaceans?

Now consider how an erotic view positions that the ocean-Other, including its inhabitants, have moral worth. We are reminded by the court cases in 2012 where PETA argued for the release of orca whales from SeaWorld under the claim that killer whales should have the same constitutional rights as people. Around the same time, philosophers, and conservation and animal behavior experts called for a Declaration of Rights for Cetaceans. In both cases, the experts claim that dolphins and whales are self-aware and sufficiently intelligent to justify the same rights as humans, as evidenced by their behavior and interaction (BBC News World 2012). Similarly to the characteristics described above for orcas, all cetaceans have large brains and share behavioral characteristics that indicate intelligence, such as complex social systems, unique communication patterns, long-term mother–calf relationships that aid in cultural and social modeling, cooperative foraging, and support in defense from predators (Parsons, Rose, and Simmonds 2004). They also suffer and experience emotions like bereavement at the loss of a loved one. The cases never went through, and a Declaration of Rights for Cetaceans has yet to pass. Despite the evidence of their sentience, what point does it make sense to grant cetaceans the right to life? And should this right be on the table for serious consideration, is the right for the cetaceans based on their own innate worth, or for their worth as prescribed by anthropocentrism?

Acknowledging that cetaceans have moral worth and deserve the same rights as humans would put an end to whaling, killing dolphins for meat, and the capturing and keeping of animals in captivity for human entertainment. Each of these factors is significantly influenced by the interwoven conversation of economics in policy. Consider Michael Sandel’s (2013) argument that commodifying whales for their economic market value are morally objectionable, because “killing a being with moral worth and an entitlement to life degrades the intrinsic value of that good, and this problem cannot be remedied by changing the way the market works as long as it is necessary for a whale to die” (Babcock 2013, p. 21). This is because the issue of valuation can no longer be summed up by how much the whale can bring in at the market, but a consideration too of its intrinsic cost. Further, Sandel explains that the act of placing a market value on a being with intrinsic moral worth degrades the worth of that being, in this case, the whale. We’ve seen this example throughout history when we’ve placed a market value on humans in slavery, prostitution, and child labor—we have othered those worthy of basic human rights when they have been reduced to a price for services expected and rendered. Recognition of innate moral worth has allowed many othered groups of people to be protected by human rights, though the fight for equity is still undergoing. So, if we place our hope in the same logic, we need to provide youth with opportunities and spaces to consider the valuation of cetaceans and make the moral worth for them a commonplace understanding. This can lead to the establishment of subsequent norms and rights that shift from *conservation* to *preservation* (Babcock 2013), a move that requires us to remove ourselves from the picture. This may seem a stretch in the Anthropocene, but our youth can be prepared to make this valuation through exposure to informal science opportunities, environments, and media like those mentioned here.

## Recommendations

While an important goal of science education is to ensure that our youth are well-rounded and informed citizens at the time of their embarkment upon the world of adulthood, what we often fail to remember is that our youth are already important decision-makers and active citizens within their communities. They come to the proverbial community table with an already established understanding of what science is, based on everyday life experiences, what they have learned in formal school settings, and through lessons of culture and family. They look to trusted adults for explanations and modeling and to community cultural institutions to help them identify what counts as science (Zimmerman and Bell 2012). In light of the discrepancy of what constitutes science among the Japanese fishing community and advocates of marine mammal protection, the inclusion of out-of-school experiences or ISE would aid youth in the development of their understanding of science. It would allow students to see that their cultural communities, family lessons, and daily experiences are valuable contributions to this understanding, which is particularly important when we consider that youth become less interested in school-based science as they grow older (e.g., Sjøberg and Schreiner 2010).

### Fostering an erotic connection in the (science) classroom

As studies have reported that meaningful and emotional engagement, what I call an erotic connection, enhances education and leads to increased well-being and proenvironmental behaviors (Yerbury, Boyd, and Weiler 2021), teachers must be prepared to foster these experiences and facilitate students in their valuation of the nature-Other and natural spaces. This requires exposure, experiences, and guided discussion because it is difficult for youth to find value in that which they are unfamiliar and unknowledgeable. One strategy is to guide students regularly, and early, through exercises that hone their senses in the classroom and within natural settings. This can be done, for example, using wondering or nature walks, sit spots for quiet outdoor observation, setting up an outdoor classroom or bringing nature indoors, and employing mindfulness strategies. This practice will not only help them to become more erotically connected to their environment, but it will also help them to become better prepared for authentic science experiences as it will bolster their ability to observe and question.

### Using environmental phenomena to explore anthropocentric value theory

Science teachers and educators should also be well versed in the anthropocentric and non-anthropocentric axiologies if they are to instill non-anthropocentric values in their students. Anthropocentric value theory is the idea that humans hold the highest value over all other forms of life, but also that all other life forms have value only inasmuch as they can be used or are important to humans. Non-anthropocentric values, then, allows for the intrinsic valuation to some non-human beings. Which non-human beings are worthy of intrinsic valuation is still up for some debate among the spectrum of environmental ethicists, though I make the argument that marine mammals fall into this category. For the sake of science educators, an important point to remember in environmental ethics, is that the responsibility for the restitution of natural resources falls squarely on the shoulders of humans. In his 1984 discussion of non-anthropocentric value theory, J. Baird Callicott stated that there is “something clearly morally wrong about this human assault on non-human forms of life and natural systems” (p. 300). This gut feeling is the crux of the argument for this type of moral–ethical investigation in science education. Youth instinctively feel some semblance of this, though many would benefit from some guidance from their teachers to begin to understand their stance on non-anthropocentric issues. Targeting environmental phenomena that capture student interest is an important entry point for bringing these conversations and investigations into the science classroom. Beyond this example with marine mammals, teachers could introduce similarly studied and charismatic mammals, such as elephants and horses or animals kept for pets, farming, in zoos or aquaria. They could even extend this argument to plants, which communicate through electrical signaling and common mycorrhizal networks, and at least in one famous case—a tree that owns itself in Athens, Georgia (Mueller et al 2011). In addition to these powerful entry points, teachers could encourage students to explore how native and indigenous communities keep their sustainable cultural practices and knowledge about the local environment relevant for the next seven generations despite loss of livelihoods and a marginalization of traditional ways of knowing through colonial exploits and the Western commercialization of science. Through consideration of perspective, culture, and history, students can begin to grapple with value differences in harvesting animals and plants as an essential and mutually beneficial generosity within indigenous cultures and an economic or recreation priority most visible in their daily lives. Through vignettes and case studies similar to the examples above, youth can begin to grapple with the meaning of sustainability and issues of human need and societal and personal values. They can extend this even further through experimentation with value ranking based on additional information or a new context.

### Ethical dilemmas in teacher preparation

There is an equally important need for ensuring that preservice teachers are prepared to navigate the waters of value judgments. Because teacher preparation programs are often not designed to equip future teachers with the tools necessary to guide their students through issues of value, they can begin to take important first steps through a focus on students’ reasoning and the interests of the students, the community, and what is important for their local environment. They can implement strategies to show preservice teachers how to take advantage of the informal learning students are doing in their communities and homes, through things like social media and movies. A key to this is the focus on relevancy and being in the world, or erotically engaging with the phenomena. Further, it is necessary to ensure that students are “aware of their own inspirations, assumptions, ethical values, and the implications of their actions” (Mueller and Zeidler 2010, p.115). To assist in this endeavor, teacher education programs can model behaviors through various techniques the preservice teachers can later use in their own classrooms. For example, teacher educators can engage students in narrative reading and writing using ethical dilemmas that prompt a shift in the way youth face them. They can ask their preservice teachers to reflect on the ethical dilemmas and valuations in their daily lives and their actions regarding them. They can use vignettes and case studies to present ethical dilemmas and situations requiring moral reasoning to encourage critical thinking and the development of value judgments. They can train them in the use and benefit of Socratic Circles and critical conversations. To take this a step further, teacher preparation programs need to foster relationships with informal science settings to model how these relationships can be used in curriculum, instruction, and assessment to benefit the learning of both science content and skills, improved well-being, and proenvironmental valuation and action.

### Integrating informal science in the classroom

Finally, and more specifically considering the marine mammal scenarios above, there is an evident need to incorporate informal science learning in our formal science classes that incorporate a type of ocean literacy that includes practices that challenge students to take off “the lab-coat of science” and be more than conscious consumers of knowledge. Nonetheless, we must not neglect the principal and essential role of the school as the main institution responsible for citizen education. This effort is to show that science education can be promoted by developing collaborative actions between formal institutions (such as schools) and informal places (natural history museums, science and technology centers, among others) and other cultural equipment. In this way, youth can be conscious consumers of those goods and services that affect the health and well-being of the ocean-Others because of their innate moral worth. This informal–formal pairing can take many forms. Though teachers and teacher educators alike readily look to and utilize museums as places where ISE traditionally occurs, these experiences do not consistently influence transformational moral and ethical science learning. Rather than focus on building relationships with informal places such as museums and science centers with the hope that students will engage in these experiences and leave transformed, integrating the type of ISE outlined here in science courses, perhaps in collaboration with informal science centers, but as part of a larger holistic science education that aims to transform students’ axiologies in a way that might inspire youth activism, could be more productive in building conscious youth than attending the informal science centers alone. Science teachers and informal science educators can work together to use a variety of authentic inquiry-based projects designed to connect students with their community and environment while gaining valuable science content knowledge and tackling ecojustice issues. Inland and coastal teachers, informal science educators, and students can collaborate to create, promote, and sustain marine science-related citizen science projects and hands-on, full-bodied exploration of their natural environments. These collaborating educators can involve youth in local socioscientific issues, like those marine mammal examples listed above, that can be explored theoretically and practically to boost ocean literacy while fostering a sense of responsible citizenry in youth for ocean-related issues. Teachers and informal science educators can create student–scientist partnerships to allow youth to gain valuable science skills and content knowledge while critically examining important scientific issues in their communities. Ideally, these actions should include authentic participation and collaboration with informal science, where students investigate legitimate issues that concern them and work through the decision-making and action process with adults (Schusler, Krasny, Peters, and Decker 2009), whether elders in their community, their teachers, or scientists. They explore their environments and these issues phenomenologically in their lifeworld, embodied and with their senses, to return to the phenomena itself, as if through the eyes of a child. They do this to develop the types of meaningful relationships and experiences that lead to action for the sake of the Other. Youth learn through these erotic experiences too how interconnected they are with the nature- and ocean-Other, and how conservation and protection of these Others allow for the conservation and protection of self.

## Conclusion

At the end of this week, I will take my students on a field trip to Deer Island to clean the beach, search for nurdles, and do a brand audit of the plastic garbage we collect. This trip was already postponed once due to the aftereffects of the tropical storm—a phenomenon many people aren’t aware of unless you live through it. The rain and wind lasted days, creating unfavorable boating conditions. While this trip is not directly related to marine mammals, I know that we may find dolphins frolicking alongside the boat along our journey this time of the year. More than that, it’s an opportunity to discuss the implications of our actions and the life cycle of our garbage until it reaches the belly of the whale, so to speak. We will be fostering our erotic relationship with the ocean-Other so that we are prepared to act for it generously. While this field trip is a far cry from what many science educators can do within a school year, these opportunities can occur more locally. We will also do this exact exercise on our local beach next week. Youth in inland areas can do this along their own waterways and aquatic environments or choose something closer to home and more relevant to local interests and phenomena. That’s the beauty of phenomenology.

Phenomenology reminds us to look for possibility and prophecy, or to look at the present to describe what is possible for the future. It considers how people perceive conditions and give meaning to experiences through perception, to analyze curriculum, and provides future directions to guide policy internationally. Phenomenology also allows educators to understand how people are brought into consciousness, or how learners become perceptive to possibilities, something Maxine Greene refers to as *wide-awakeness* (Greene 1978). Greene argues for the integration of such work and activities in the curriculum to promote awareness for the quest for meaning or wide-awakeness, “to move others to elevate their lives by a ‘conscious endeavor,’ to arouse others to discover—each in his or her own terms—what it means to ‘live deliberately’” (1977, p. 120), or what Van Manen (1984) would call “living life deeply.” Part of the phenomenological method, after all, is that the being “stands in the fullness of life, in the midst of the world of living relations and shared situations” (Van Manen 1984, p. 40). Such a curriculum using ISE will encourage each student to bracket out the empirical world to reveal their unique primitive self and provoke the type of reflection necessary to enable them to pay full attention to life. Further, the use of phenomenology with ISE within the science classroom may encourage students to challenge what is taken for granted (the *Status Quo*), highlight the shadows (what goes unnoticed and unchecked), examine presuppositions, and think critically about the differences in the primitive world they experience bodily compared to the world they grew into.

Through the ethic, we can recognize that generosity is a better way to act in our relationship to the Other, rather than through violence, or subjectivity and oppression. If science teachers and informal science educators encouraged an erotic ethic within their practice, the dominant view of patriarchy might dissolve, creating a place for freedom from oppression. This freedom will not only occur for individual students and science educators but also freedom for the Other through erotic generosity. As students experience the world in the flesh, they will allow the Other (e.g., ocean and inhabitants) to be free from objectification, encouraging them (youth) to act for the conservation and care of the Other.
